# Low Frustration Tolerance, Childhood Perfectionism, and Mental Health Problems in Peruvian Children: A Network Analysis

**DOI:** 10.3390/children13101411

**Published:** 2026-10-15

**Authors:** Roger Angulo-Salas, Jonatan Baños-Chaparro, María Ortiz-Romaina

**Affiliations:** 1 Facultad de Ciencias de la Salud, Universidad Católica Sedes Sapientiae, Lima 15302, Peru; rjangulo@ucss.edu.pe (R.A.-S.); 2022102083@ucss.pe (M.O.-R.); 2 Programa Académico de Psicología, Facultad de Ciencias de la Salud, Universidad Privada Norbert Wiener, Lima 15046, Peru

**Keywords:** children, low frustration tolerance, childhood perfectionism, mental health problems, internalizing problems, externalizing problems, attention problems, network analysis

## Abstract

**Highlights:**

**What are the main findings?**

- Low frustration tolerance and reaction to failure showed the highest observed expected influence values within the six-node dimension-level network.
- The strongest edges involved reaction to failure–internalizing problems, low frustration tolerance–externalizing problems, perfectionistic self-demands–reaction to failure, and low frustration tolerance–reaction to failure.

**What are the implications of the main findings?**

- Low frustration tolerance and reaction to failure may represent relevant processes for further longitudinal and school-based preventive research.
- Future school-based prevention studies could evaluate strategies focused on emotional regulation, adaptive responses to error, and self-critical perfectionistic responses.

**Abstract:**

**Background/Objectives:** Childhood mental health problems are often organized into interrelated emotional, attentional, and behavioral domains. However, little is known about how low frustration tolerance and childhood perfectionism are connected with these domains in school-aged children. This study aimed to estimate the network structure of low frustration tolerance, childhood perfectionism, and mental health problems in Peruvian children aged 8 to 12 years. **Methods:** A cross-sectional study was conducted with 707 Peruvian children from public and private schools in Lima, Peru. The assessed variables were low frustration tolerance, perfectionistic self-demands, reaction to failure, internalizing problems, attention problems, and externalizing problems. A non-regularized Gaussian graphical model was estimated using Spearman correlations. Expected influence was calculated to identify central nodes. Network accuracy and stability were evaluated using nonparametric bootstrap and case-dropping procedures. Network comparisons were performed according to sex and school type. **Results:** In the full-sample dimension-level network, eight edges were identified, all of which were positive. Low frustration tolerance and reaction to failure showed the highest observed expected influence values. The strongest associations were observed between reaction to failure and internalizing problems, low frustration tolerance and externalizing problems, perfectionistic self-demands and reaction to failure, and low frustration tolerance and reaction to failure. Expected influence showed adequate case-dropping stability. Exploratory network comparisons identified the largest observed sex difference in the association between reaction to failure and internalizing problems, whereas global strength was descriptively higher in public than private schools; however, these omnibus group differences did not remain statistically significant after correction for multiple grouping comparisons. **Conclusions:** Low frustration tolerance and reaction to failure emerged as highly connected dimensions within the estimated network. These findings identify candidate processes for further longitudinal and preventive research focused on emotional regulation and adaptive responses to failure while not implying causal or intervention effects.

## 1. Introduction

Mental health difficulties often become apparent during childhood and can disrupt day-to-day functioning and later development, yet timely identification and care remain unavailable for many affected children [1,2,3]. Middle childhood is especially relevant because emotional, cognitive, and social capacities are still developing while interactions with family, school, and peers become more complex [4,5,6]. From a dimensional perspective, child mental health difficulties are commonly described in internalizing, externalizing, and attention-related domains. Internalizing problems include sadness, anxiety, worry, and withdrawal; externalizing problems include aggressive, oppositional, impulsive, or rule-breaking behaviors; and attention problems involve concentration difficulties, distractibility, restlessness, and behavioral control [7,8,9,10,11]. Examining these domains together with broader emotional and self-evaluative processes may clarify how they are conditionally related during childhood.

Low frustration tolerance is one process that may be relevant to this organization. Frustration arises when expected goals or rewards are blocked, and difficulty tolerating such experiences may be expressed through anger, impulsive responding, difficulty waiting, or intense reactions to mistakes [12,13]. In the present study, low frustration tolerance is used in the specific sense operationalized by the Frustration Tolerance Scale: difficulty controlling anger, tolerating adverse or frustrating situations, and regulating impulsive responses. This construct overlaps with, but is not equivalent to, irritability, which more broadly reflects a heightened propensity toward anger and temper outbursts, or distress tolerance, which refers to the perceived capacity to withstand aversive emotional states [14]. Conceptual and developmental work on emotion regulation, irritability, and disruptive behavior further indicates that these processes are related to internalizing, externalizing, and attentional difficulties in young people [15,16,17,18,19,20]. Accordingly, low frustration tolerance may be meaningfully connected with several mental health domains, particularly externalizing difficulties.

Perfectionism represents a second potentially relevant process. It is multidimensional and includes both the pursuit of high standards and negative self-evaluative responses when those standards are not achieved [21,22,23,24]. In childhood, perfectionistic self-demands reflect elevated internal expectations regarding performance, whereas reaction to failure captures negative emotional responses to mistakes, errors, or perceived failure. Evidence in young people indicates that perfectionistic concerns are more consistently associated with anxiety and depressive symptoms than perfectionistic strivings [25], while childhood perfectionism has also been related to internalizing and externalizing difficulties [26,27]. Thus, reaction to failure may be especially connected with internalizing problems, whereas its association with perfectionistic self-demands may reflect the link between high standards and negative responses when expectations are not met [28].

Network analysis provides a framework for examining these constructs simultaneously by estimating conditional associations among symptoms or dimensions rather than considering each relationship in isolation [29,30,31]. Within this framework, centrality indices can describe the relative connectedness of nodes within the estimated network [32,33]. Previous network studies in children and adolescents have identified inattention, worry, irritability, and behavioral problems as relevant components of psychopathology networks [34,35,36]. However, prior research has largely examined these domains separately, and evidence is lacking on how low frustration tolerance and distinct dimensions of childhood perfectionism are jointly connected with internalizing, attention, and externalizing problems. This gap is particularly evident in Latin American samples. The present study therefore extends previous work by integrating these emotional, self-evaluative, and mental health dimensions within a single dimension-level network in Peruvian schoolchildren.

Sex and school type were also examined as grouping variables. Differences by sex have been reported in developmental patterns of internalizing and externalizing problems and in the expression of irritability [10,19], providing a rationale for examining whether the estimated network differs between boys and girls. School type was considered as a contextual grouping variable because children develop within educational environments that may differ in organization and resources [4,5,6]; however, it was not treated as a validated proxy for socioeconomic position, school climate, academic pressure, or access to psychological services. Given the limited evidence for network differences across these groups, comparisons by sex and school type were considered exploratory. Based on the literature, we expected positive conditional associations between low frustration tolerance and externalizing problems, between reaction to failure and internalizing problems, and between perfectionistic self-demands and reaction to failure. We also expected low frustration tolerance and reaction to failure to show comparatively high connectivity, without specifying a rank ordering among nodes. Accordingly, the study aimed to estimate a six-node dimension-level network of low frustration tolerance, perfectionistic self-demands, reaction to failure, internalizing problems, attention problems, and externalizing problems in Peruvian children aged 8 to 12 years from public and private schools in Lima, and to explore network differences by sex and school type.

## 2. Materials and Methods

### 2.1. Participants and Procedure

A cross-sectional, non-experimental observational study was conducted using an analytical approach based on psychological network analysis. The sample comprised 707 Peruvian children of both sexes, aged 8 to 12 years, enrolled from third to sixth grade of primary education in four schools in Lima, Peru (two public and two private), encompassing approximately 20 classrooms. The schools were recruited by convenience according to institutional authorization and feasibility of access, and recruitment was conducted at the classroom level. Students were eligible if they were enrolled in the participating schools, had written authorization from a parent or legal guardian, and provided assent. Students who did not complete the questionnaires adequately, provided incomplete information, or withdrew were not included in the analytic sample. The protocol also contemplated nonparticipation when a previously known medical, psychological, or learning condition was considered to interfere with the child’s ability to understand or complete the questionnaires under the standard classroom procedure. Such conditions were not diagnosed as part of this study. The available study records did not retain a separate count of exclusions attributable to this criterion.

Sample-size adequacy for the network model was examined with an iterative Monte Carlo procedure implemented in the powerly package in R [37]. The simulated Gaussian graphical model contained six nodes and assumed an edge density of 0.40, representing a moderately connected structure in which some, but not all, conditional associations were present. The performance target was a sensitivity of 0.60 (the proportion of true edges recovered), and the probability of reaching at least that level of sensitivity was set to 0.99. Thirty replications were evaluated across candidate sample sizes. The resulting recommendation was a minimum of 300 participants; the final analytical sample comprised 707 children.

For the subgroup analyses, the sample sizes were 359 boys and 348 girls, and 332 children from private schools and 375 from public schools. Each subgroup therefore exceeded the minimum sample size estimated for the six-node network itself. However, the original Monte Carlo calculation was designed to evaluate sample adequacy for network estimation rather than the statistical power of between-group Network Comparison Tests; accordingly, no separate a priori power analysis was conducted specifically for the NCT.

For data collection, authorization was first requested from the authorities of the four participating schools, who were informed about the study objectives and assessment conditions. Written informed consent forms were distributed to parents or legal guardians, and assessment dates were coordinated with teachers. According to the records available for the present report, the exact numbers of students initially approached, consent forms distributed or returned, and students who declined participation were not retained in a form that permits calculation of a participation rate; the final analytic sample comprised 707 children who met the inclusion requirements and completed the assessment. Questionnaires were administered collectively during authorized school hours in October and November 2025, while each child completed his or her own questionnaire. Evaluators read standardized instructions aloud and remained in the classroom to clarify procedural questions or wording when requested; clarification was limited to facilitating comprehension and did not involve suggesting responses. Students were reminded that participation was voluntary, confidential, and anonymous and that they could withdraw at any time. After administration, questionnaires were reviewed to identify incomplete or inconsistent responses.

Table 1 shows that the mean age of the primary school students was 10.27 years (SD = 1.22). Participation was balanced by sex, with 50.8% boys and 49.2% girls. Regarding school type, 46.9% of participants attended private schools and 53.1% attended public schools. School type was coded solely from the institution’s administrative status (public vs. private) and was used as an observed institutional grouping variable; it was not interpreted as a proxy for socioeconomic position, school climate, academic pressure, or access to psychological services.

### 2.2. Ethical Considerations

The study protocol was approved by the Research Ethics Committee of the Faculty of Health Sciences of the Universidad Católica Sedes Sapientiae through Approval Letter No. CE-2198, dated 1 October 2025. As the participants were minors, written informed consent was obtained from parents or legal guardians, and assent was obtained from the children. Participation was voluntary, anonymous, and confidential.

### 2.3. Measures

Confirmatory factor analyses were conducted in the present sample using the weighted least squares mean and variance adjusted (WLSMV) estimator, treating item responses as ordinal. Model fit was evaluated using the comparative fit index (CFI), Tucker–Lewis index (TLI), root mean square error of approximation (RMSEA), and standardized root mean square residual (SRMR), using conventional thresholds (CFI and TLI > 0.90; RMSEA and SRMR < 0.08) as descriptive guides to model fit.

Before comparing networks by sex and school type, measurement invariance of the three instruments was examined using multigroup confirmatory factor analysis with the WLSMV estimator. Configural, threshold, metric, and scalar invariance models were sequentially evaluated, with invariance assessed primarily from changes in comparative fit indices across increasingly constrained models.

#### 2.3.1. Low Frustration Tolerance

Low frustration tolerance was assessed using the Frustration Tolerance Scale, derived from a subscale of the Bar-On Emotional Quotient Inventory [38], originally adapted by Oliva et al. [39] and validated in Peruvian children by Ventura-León et al. [40]. The instrument has a one-dimensional structure composed of 8 items assessing difficulties controlling anger, tolerating adverse or frustrating situations, and regulating impulsive responses. In this study, the label low frustration tolerance refers specifically to the score operationalized by this instrument and should not be interpreted as interchangeable with broader constructs such as distress tolerance or with the broader clinical phenotype of irritability. Items are answered using a five-point Likert scale ranging from 1 = never to 5 = always. Total scores are obtained by summing the items, with possible scores ranging from 8 to 40, where higher scores indicate greater low frustration tolerance. In the present sample, the one-dimensional model showed adequate fit indices (CFI = 0.98, TLI = 0.98, SRMR = 0.04, RMSEA = 0.06, 90% CI [0.04, 0.07]). Internal consistency was satisfactory (α = 0.84; ω = 0.81).

#### 2.3.2. Childhood Perfectionism

Childhood perfectionism was assessed using the Childhood Perfectionism Scale, originally developed by Oros [41] and validated in Peruvian children by Ventura-León et al. [42]. The instrument has a two-dimensional structure comprising 16 items distributed into two dimensions: perfectionistic self-demands, consisting of items 1 to 8 and referring to high internal demands related to performance and personal achievement, and reaction to failure, consisting of items 9 to 16 and referring to negative emotional responses to mistakes, errors, or failure. Items are answered using a three-point Likert scale: 1 = I do not think so/no, 2 = I think so sometimes/sometimes, and 3 = I think so/yes. Scores for perfectionistic self-demands and reaction to failure were calculated separately, with each subscale ranging from 8 to 24; higher scores indicate higher levels of the corresponding dimension. The total score was not used in the network analysis. In the present sample, the two-dimensional model showed acceptable fit indices (CFI = 0.94, TLI = 0.93, SRMR = 0.07, RMSEA = 0.07, 90% CI [0.06, 0.08]). Regarding internal consistency, perfectionistic self-demands showed satisfactory coefficients (α = 0.89; ω = 0.84), as did reaction to failure (α = 0.86; ω = 0.78).

#### 2.3.3. Mental Health Problems

Mental health problems were assessed using the 17-item Pediatric Symptom Checklist, youth self-report version (PSC-17-Y), originally developed by Gardner et al. [43] as a brief version of the Pediatric Symptom Checklist with psychosocial problem subscales. The Peruvian adaptation reported by Tirado-Hurtado et al. [44] included participants aged 7 to 17 years; however, that validation administered the parent-report PSC-17 to children aged 7 to 11 years and the self-report PSC-17-Y to adolescents aged 12 to 17 years. Therefore, the Peruvian validation does not provide direct age-specific evidence for self-administration of the PSC-17-Y in children aged 8 to 11 years. In the present study, all participants completed the self-report form. The instrument has a three-dimensional structure composed of 17 items distributed across internalizing problems (5 items), attention problems (5 items), and externalizing problems (7 items). Items are answered using a three-point Likert scale: 0 = never, 1 = sometimes, and 2 = often. Total scores range from 0 to 34, with higher values indicating greater risk of psychosocial impairment. To provide psychometric evidence within the present sample, the three-dimensional model was evaluated and showed adequate fit indices (CFI = 0.93, TLI = 0.92, SRMR = 0.06, RMSEA = 0.05, 90% CI [0.04, 0.05]). Internalizing problems showed acceptable internal consistency (α = 0.76; ω = 0.71), while attention problems (α = 0.71; ω = 0.70) and externalizing problems (α = 0.73; ω = 0.71) showed moderate coefficients. These sample-specific results support score use in the current analyses but do not substitute for age-specific validation of the PSC-17-Y in younger children; this issue is considered in the limitations.

### 2.4. Data Analysis

Analyses were performed in R version 4.3.2 (R Foundation for Statistical Computing, Vienna, Austria) within the RStudio integrated development environment (Posit Software, PBC, Boston, MA, USA); the exact IDE version was not retained. Screening of the final analytical dataset identified no missing responses (0/707 participants; 0%), so no imputation or other missing-data procedure was required before network estimation. Means and standard deviations were used to summarize the study variables. Potential node redundancy was examined with the goldbricker function in networktools (v. 1.5.0), using a >25% threshold and *p* = 0.05 [45,46].

The undirected network estimation was performed in four consecutive stages. First, a non-regularized model was fitted using the estimateNetwork function from the bootnet package (v. 1.5.3), implementing the ggmModSelect algorithm with Spearman correlations. Spearman correlations were selected because the six study scores were derived from ordinal-response instruments and could depart from normality; this correlation is appropriate for monotonic associations and is less sensitive to distributional departures [47]. The ggmModSelect procedure evaluated a graphical-lasso tuning path with 100 tuning-parameter values to generate candidate network structures, which were subsequently refitted without regularization. Model selection used BIC (equivalent here to EBIC with γ = 0), with stepwise edge-addition and edge-removal search enabled until no further improvement in the information criterion was obtained. A non-regularized model-selection approach was considered appropriate because the network was low-dimensional (six nodes; 15 possible edges) relative to the sample size (n = 707), allowing model sparsity to be selected through the information criterion while avoiding shrinkage of retained edge weights [48,49]. The network was visualized using qgraph (v. 1.9.5) and the Fruchterman–Reingold layout [50,51].

In the second stage, expected influence was calculated using the centrality function from qgraph as a descriptive index of the relative connectedness of each node, taking the sign and magnitude of its edges into account [50]. Graph density was defined as the proportion of observed edges among all 15 possible edges in the six-node network. In addition, the mean edge weight across all possible node pairs was calculated as a separate descriptive quantity. Because expected influence values were not subjected to pairwise centrality-difference testing, their ordering was interpreted descriptively rather than as evidence that nodes differed significantly in centrality.

In the third stage, uncertainty in edge estimates and stability of expected influence were evaluated separately. Edge-weight precision was examined with 1000 nonparametric bootstrap samples in bootnet (v. 1.5.3), from which 95% confidence intervals were obtained [48]. Expected-influence stability was assessed with case-dropping bootstrap by repeatedly re-estimating the network after removing increasing proportions of participants. The correlation stability coefficient (CS) represents the largest proportion of cases that can be dropped while, with 95% probability, retaining a correlation of at least 0.70 between the original and subset centrality estimates; CS values above 0.25 are considered acceptable, and values of approximately 0.50 or higher are preferred [52,53].

Finally, network comparisons were conducted by sex and school type. Descriptive similarity between two group networks was quantified by correlating the vectors of corresponding unique edge weights. Formal comparisons used the Network Comparison Test (NCT) implemented in the NetworkComparisonTest package with 1000 random permutations [54]. The structural invariance statistic (M) was defined as the maximum absolute difference between corresponding edge weights in the two networks, whereas the global-strength statistic compared the sums of absolute edge weights. Because two grouping variables were examined, Holm correction was applied across the sex and school-type analyses separately for structural invariance and global strength; both unadjusted and adjusted *p* values are reported. Nonsignificant results were interpreted as a lack of detected evidence for group differences rather than as proof of network equivalence.

Given the subgroup sample sizes and the absence of an a priori power analysis specifically designed for the NCT, an additional simulation-based sensitivity analysis was conducted. Using the observed subgroup-specific means and covariance structures and the actual subgroup sample sizes, 250 datasets were simulated. For each simulated dataset, network comparisons by sex and school type were repeated using the same NCT specifications as in the main analyses, with 1000 permutations. Empirical detection rates were calculated for network structure, global strength, and, for the sex comparison, the reaction-to-failure–internalizing-problems edge; detection rates after Holm correction were also calculated for the global structure and global strength tests.

## 3. Results

### 3.1. Global Network Properties

The full-sample dimension-level network contained 8 of the 15 possible edges, corresponding to a graph density of 0.53. All eight retained edges were positive. The mean edge weight across all possible node pairs was 0.142.

### 3.2. Local Network Properties

Regarding descriptive statistics, Table 2 shows that the highest mean and standard deviation were observed for low frustration tolerance (M = 16.78, SD = 5.85), whereas the lowest values were observed for externalizing problems (M = 2.61, SD = 2.16). The redundancy analysis did not identify equivalent node pairs, suggesting the absence of relevant topological overlap among the variables included.

**Figure 1:**
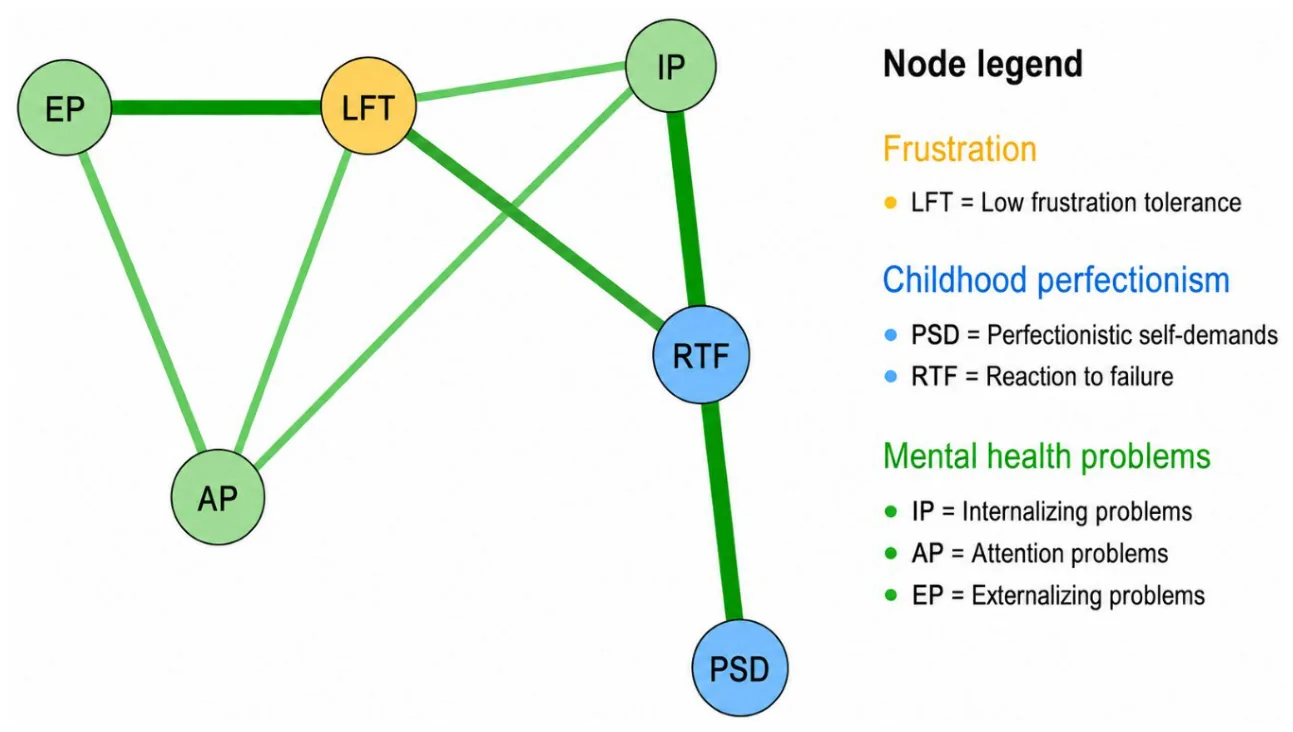
Dimension-level network of low frustration tolerance, childhood perfectionism, and mental health problems in Peruvian children. Abbreviations: LFT = low frustration tolerance; PSD = perfectionistic self-demands; RTF = reaction to failure; IP = internalizing problems; AP = attention problems; EP = externalizing problems. Green lines indicate positive conditional associations; thicker and darker lines represent stronger associations.

Regarding local properties, the highest observed expected influence values were found for low frustration tolerance (EI = 1.02) and reaction to failure (EI = 0.99), followed by internalizing problems (EI = 0.75). These values are interpreted descriptively because pairwise differences between centrality estimates were not formally tested. Regarding the strongest conditional associations, Figure 1 shows that the largest edge weights emerged between reaction to failure and internalizing problems (r = 0.38), low frustration tolerance and externalizing problems (r = 0.36), perfectionistic self-demands and reaction to failure (r = 0.34), and low frustration tolerance and reaction to failure (r = 0.28).

### 3.3. Accuracy and Stability of the Network Structure

**Figure 2:**
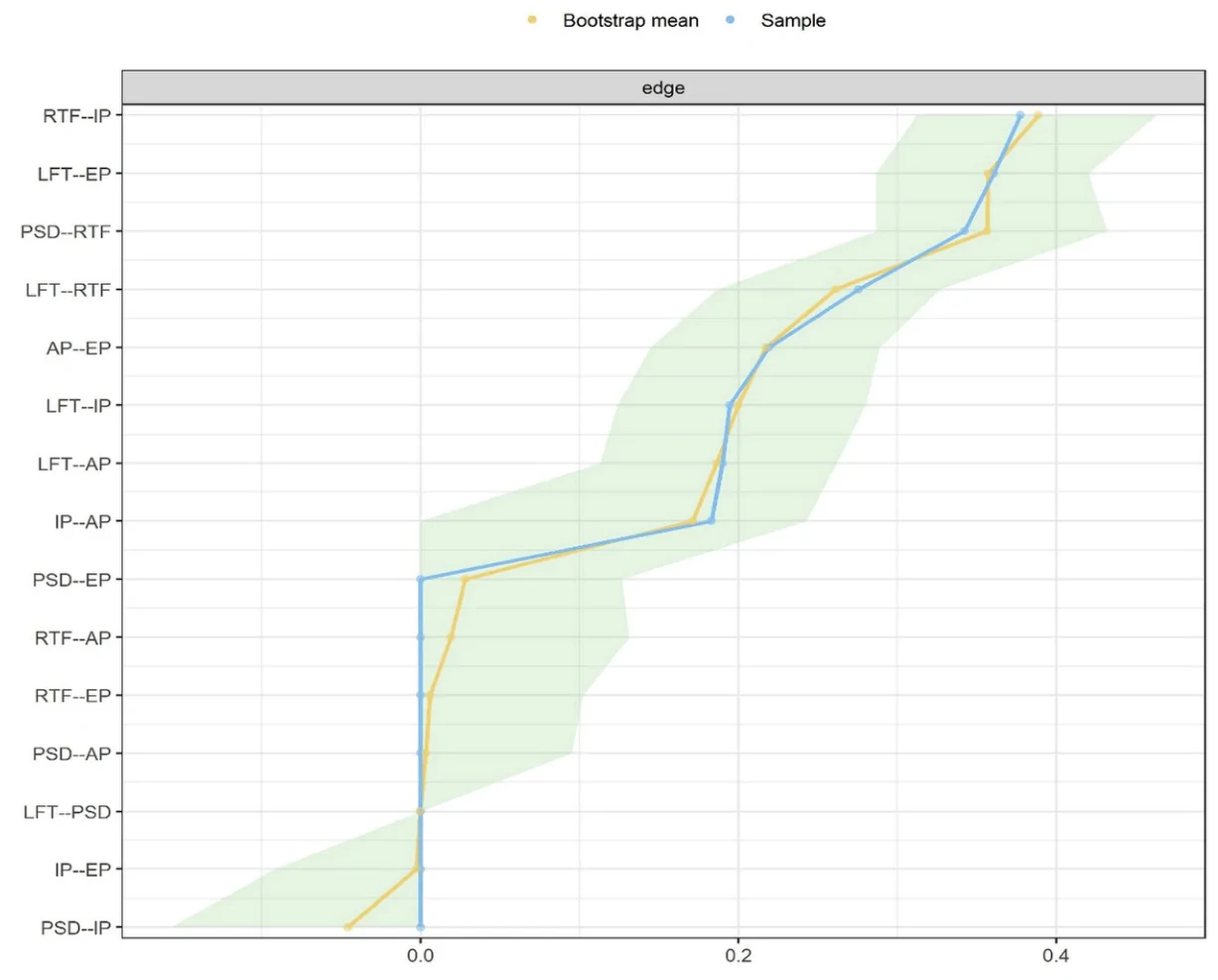
Nonparametric bootstrap 95% confidence intervals for estimated edge weights in the dimension-level network. Abbreviations: LFT = low frustration tolerance; PSD = perfectionistic self-demands; RTF = reaction to failure; IP = internalizing problems; AP = attention problems; EP = externalizing problems. The blue line represents the sample edge estimates, the yellow line represents the bootstrap means, and the green shaded areas represent the 95% bootstrap confidence intervals.

**Figure 3:**
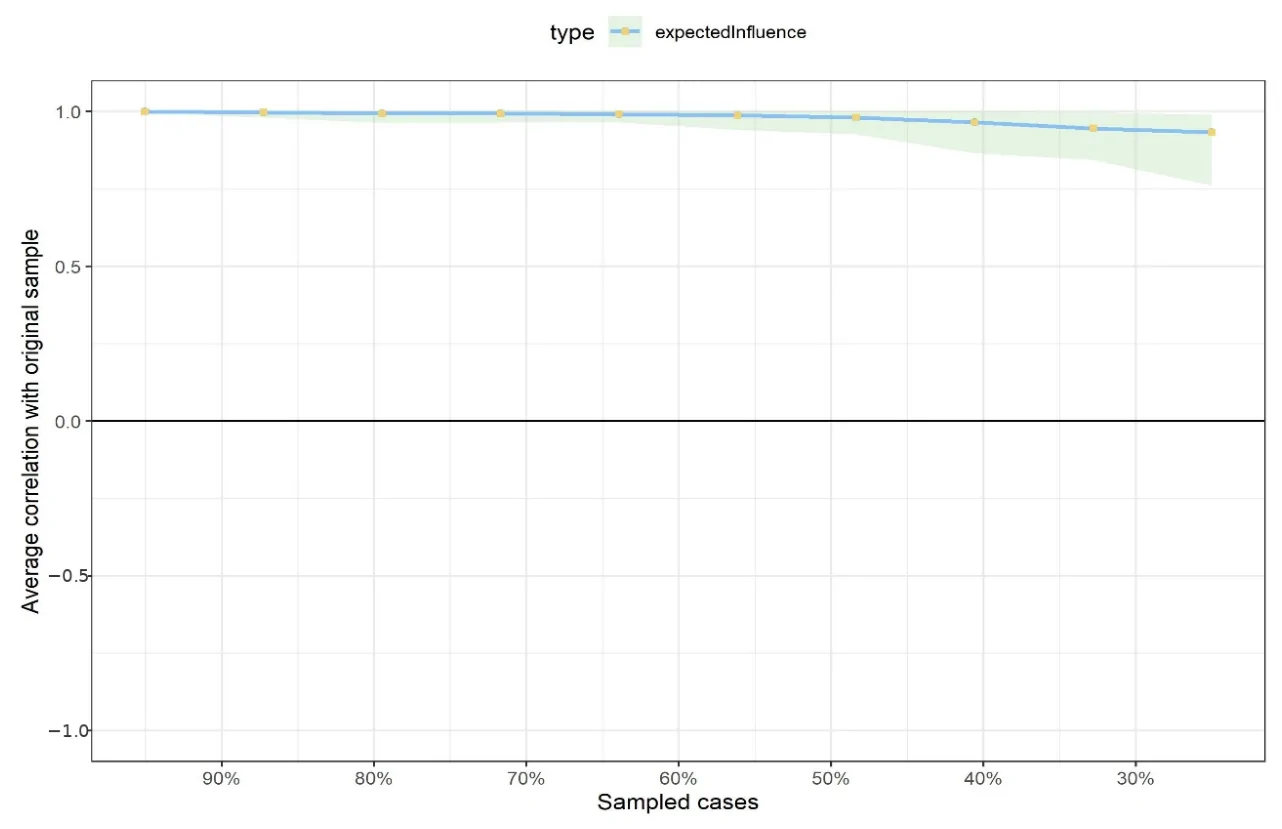
Stability of the expected influence centrality index.

Figure 2 presents edge-weight accuracy based on nonparametric bootstrap confidence intervals. The strongest edges showed narrower estimates, whereas weaker connections showed greater uncertainty. Figure 3 presents the case-dropping stability of expected influence. The correlation stability coefficient was CS(cor = 0.70) = 0.75, corresponding to the highest case-dropping proportion evaluated and indicating high stability of the expected-influence estimates under case dropping.

### 3.4. Comparative Analysis by Sex and School Type

**Figure 4:**
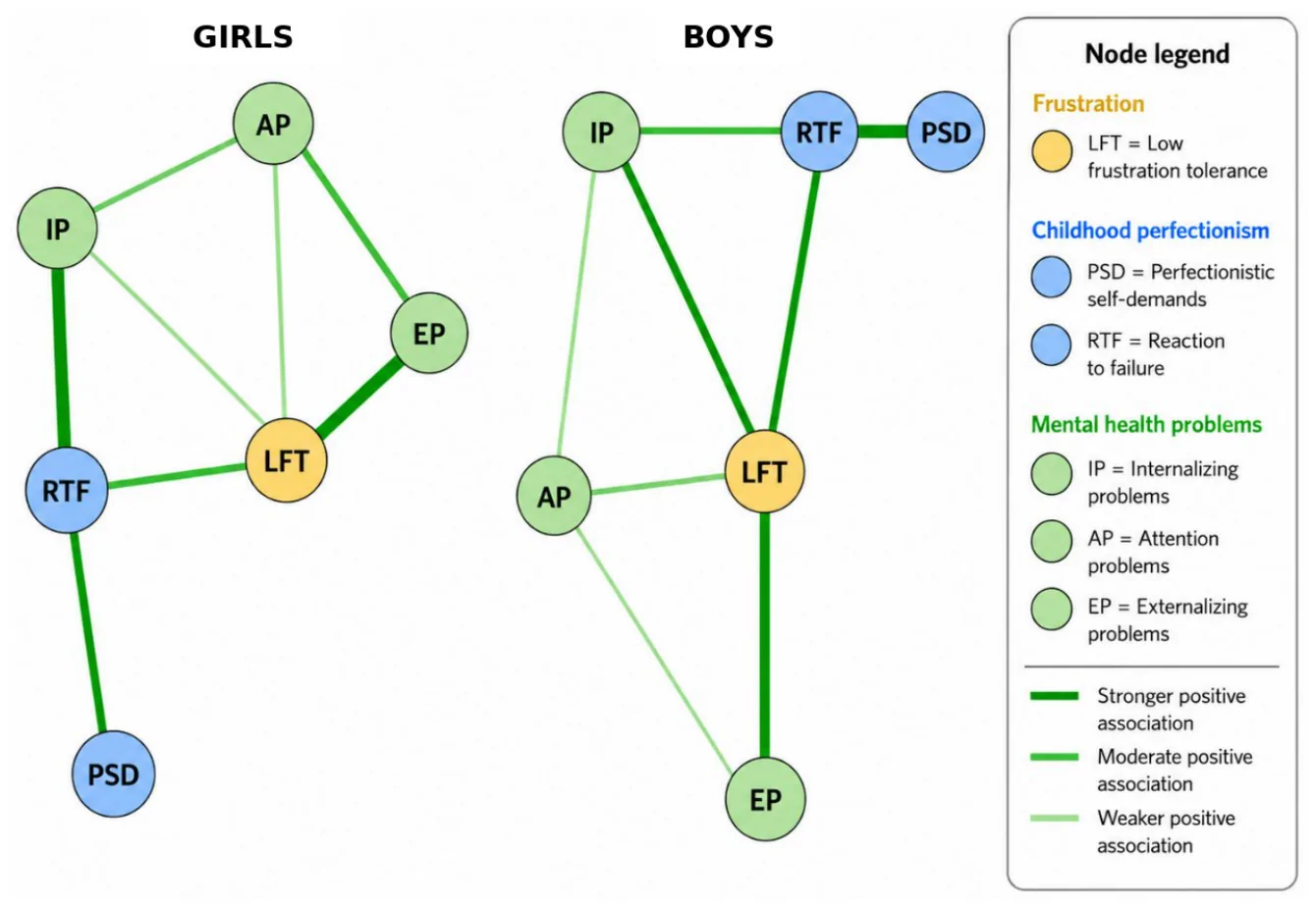
Comparative dimension-level network structures by sex (girls and boys). Abbreviations: LFT = low frustration tolerance; PSD = perfectionistic self-demands; RTF = reaction to failure; IP = internalizing problems; AP = attention problems; EP = externalizing problems.

Figure 4 reports the network comparison by sex. The correlation between the corresponding edge-weight vectors was high (r = 0.92). The structural invariance test yielded M = 0.196 (unadjusted *p* = 0.032; Holm-adjusted *p* = 0.064), so the observed omnibus structural difference did not remain statistically significant after correction across the two grouping comparisons. The largest observed edge difference involved reaction to failure and internalizing problems, with a stronger association among girls (edge weight = 0.455) than boys (edge weight = 0.259; observed difference = 0.196). No statistically significant difference in global strength was detected between the female and male networks (2.19 vs. 1.97, respectively; S = 0.214, unadjusted *p* = 0.205; Holm-adjusted *p* = 0.205).

**Figure 5:**
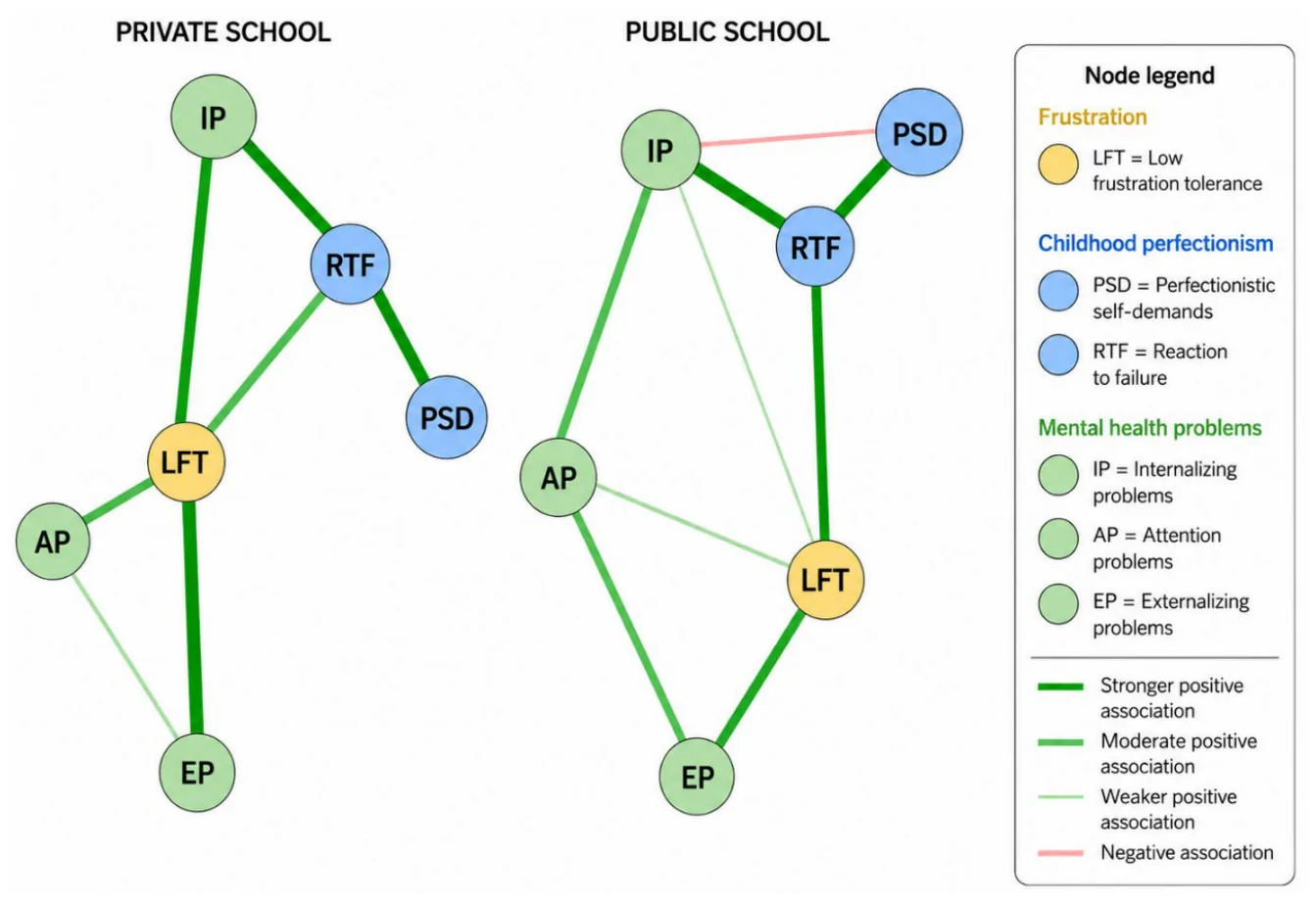
Comparative dimension-level network structures by school type (private and public schools). Abbreviations: LFT = low frustration tolerance; PSD = perfectionistic self-demands; RTF = reaction to failure; IP = internalizing problems; AP = attention problems; EP = externalizing problems.

Figure 5 shows the comparison by school type. The correlation between the corresponding edge-weight vectors was high (r = 0.81). The public-school network included one negative conditional association between perfectionistic self-demands and internalizing problems, whereas the statement that eight retained edges were positive refers only to the full-sample network. The structural invariance test was not statistically significant (M = 0.161, unadjusted *p* = 0.147; Holm-adjusted *p* = 0.147), indicating that no statistically significant structural difference was detected between the public- and private-school networks. Global strength was descriptively higher in the public-school network (2.49) than in the private-school network (2.09), with an observed difference of 0.403; however, the unadjusted *p* = 0.029 did not remain statistically significant after Holm correction (adjusted *p* = 0.058).

Measurement invariance analyses supported the comparability of the measurement models across sex and school type. Across the Frustration Tolerance Scale, Childhood Perfectionism Scale, and PSC-17-Y, progressively constrained models showed only small changes in CFI, RMSEA, and SRMR, supporting configural, metric, and scalar invariance. Detailed results are presented in Supplementary Table S1.

The simulation-based sensitivity analysis indicated heterogeneous detection rates across the network comparison statistics. For the sex comparison, the empirical detection rate was 0.580 for network structure and 0.120 for global strength, decreasing to 0.520 and 0.088, respectively, after Holm correction. The specific reaction-to-failure–internalizing-problems edge showed a substantially higher detection rate of 0.844. For school type, the detection rates were 0.628 for network structure and 0.300 for global strength, decreasing to 0.572 and 0.196, respectively, after Holm correction. These results indicate greater sensitivity for detecting the specific edge difference than for detecting global-strength differences. Detailed results are presented in Supplementary Table S2.

## 4. Discussion

### 4.1. Main Findings and Network Structure

The present study examined conditional associations among low frustration tolerance, two dimensions of childhood perfectionism, and three domains of mental health problems in Peruvian schoolchildren. Because the analysis was cross-sectional and based on six composite dimension scores, the resulting network should be interpreted as a parsimonious representation of between-domain conditional associations rather than as evidence of symptom-level dynamics, temporal ordering, or causal processes. Within this scope, the findings extend dimensional and transdiagnostic perspectives by considering emotional regulation, perfectionistic responses, and internalizing, attentional, and externalizing difficulties within the same analytical framework [7,8,29,30,31,34,36].

Low frustration tolerance and reaction to failure displayed the highest observed expected-influence values, although their centrality estimates were not statistically compared. Their comparatively high connectivity is theoretically plausible: frustration-related dysregulation and irritability have been associated with a broad range of emotional, attentional, and behavioral difficulties [19,20], whereas perfectionistic concerns and negative reactions to mistakes are consistently associated with psychological distress [23,25,28]. Accordingly, these dimensions are best viewed as potentially relevant correlates for hypothesis generation and future longitudinal research, not as established intervention targets.

The conditional association between low frustration tolerance and externalizing problems is consistent with the psychological proximity between difficulty regulating frustration and outwardly expressed behavioral difficulties. Children who report greater problems managing frustrating situations may also report more impulsive, oppositional, or dysregulated behavior; however, the present data do not establish the direction of this association. Previous work linking irritability and emotion dysregulation with externalizing manifestations provides convergent support for this interpretation [19,20].

Reaction to failure was strongly conditionally associated with internalizing problems. This pattern is compatible with cognitive–behavioral models of perfectionism, in which self-critical responses to mistakes and achievement-contingent self-evaluation are associated with distress [28], and with meta-analytic evidence showing stronger associations of perfectionistic concerns than perfectionistic strivings with anxiety and depressive symptoms [23,25]. In the present study, this edge should therefore be interpreted as a contemporaneous association between composite dimensions rather than as a pathway through which perfectionism produces internalizing problems.

The remaining prominent associations also have a coherent psychological interpretation. Perfectionistic self-demands were conditionally related to reaction to failure, consistent with the distinction between striving for high standards and responding negatively when those standards are not met [23,24]. Low frustration tolerance was likewise related to reaction to failure, suggesting that emotional reactivity to blocked goals and negative responses to mistakes co-occur within the assessed dimensions. These relationships may be particularly relevant in school settings, where performance evaluation and errors are frequent, but the present design does not establish whether one process precedes or intensifies the other.

Bootstrap analyses addressed uncertainty in complementary ways: nonparametric intervals described the precision of edge estimates, whereas case-dropping bootstrap assessed the stability of expected influence. The comparatively stable expected-influence estimates support cautious interpretation of the descriptive connectivity pattern, but neither bootstrap procedure establishes causal direction or statistically significant differences between centrality estimates [52,53].

Group comparisons were exploratory. The largest observed sex-related edge difference involved reaction to failure and internalizing problems, with a stronger conditional association among girls (0.455) than boys (0.259; observed difference = 0.196). Nevertheless, the omnibus structural comparison did not remain statistically significant after Holm correction, so this pattern should not be interpreted as a confirmed sex difference or used to infer sex-specific psychological mechanisms. Replication in independent samples is needed before drawing developmental or clinical conclusions.

For school type, global strength was descriptively higher in public schools (2.49) than in private schools (2.09; observed difference = 0.403), but this difference did not remain statistically significant after Holm correction. School type was only an observed administrative classification; therefore, the pattern cannot be attributed to socioeconomic position, school climate, academic pressure, family resources, parental expectations, or access to psychological services. These contextual characteristics should be measured directly in future studies if their role in network differences is to be evaluated.

### 4.2. Practical Implications for School Mental Health

The findings are most appropriately used to generate hypotheses for school mental health research rather than to identify confirmed intervention targets. The observed connectivity of low frustration tolerance and reaction to failure suggests that future longitudinal and experimental studies could examine whether emotional regulation, frustration management, adaptive responses to error, and self-critical reactions to failure are relevant to subsequent emotional or behavioral adjustment. Evidence from intervention or temporal designs would be required before recommending changes to these processes as mechanisms for prevention.

### 4.3. Strengths, Limitations, and Future Directions

This study has several strengths, including a relatively large sample of schoolchildren, participation from both public and private schools, psychometric evaluation of the measures in the current sample, assessment of measurement invariance across the grouping variables, and separate evaluation of edge accuracy and centrality stability. The dimension-level network also provided a parsimonious way to examine conditional associations among theoretically distinct domains within a single model.

Several limitations qualify the interpretation of the findings. First, the cross-sectional design precludes causal and temporal inferences; the estimated edges represent contemporaneous conditional associations only. Longitudinal and temporal network studies with repeated measurements are a priority for examining whether these associations persist over time and whether changes in one domain precede changes in another. Second, convenience sampling from four schools in Lima limits external validity. The findings are most applicable to schoolchildren with characteristics and educational contexts similar to those represented here and should not be generalized to all children in Peru or Latin America. The inability to reconstruct the number initially approached, refusals, and exclusions by reason further limits assessment of potential selection bias.

Third, all variables were assessed by child self-report during the same assessment session. Shared method, response style, comprehension, and situational factors may therefore have increased covariance among measures, creating the possibility of common-method variance. Future studies should combine child reports with parent and teacher ratings, behavioral tasks, or observational indicators. This limitation is especially relevant for the PSC-17-Y because the available Peruvian validation used parent report for children aged 7–11 years and self-report for adolescents aged 12–17 years; consequently, self-administration in participants aged 8–11 years extends beyond the age range with direct local validation, despite the acceptable factor structure and internal consistency observed in the present sample.

## 5. Conclusions

This study provides a dimension-level description of how low frustration tolerance, childhood perfectionism, and mental health problems were conditionally associated in a sample of Peruvian schoolchildren. Low frustration tolerance and reaction to failure showed the highest observed expected-influence values, and prominent conditional associations linked reaction to failure with internalizing problems and low frustration tolerance with externalizing problems. These findings characterize the structure of the observed cross-sectional data and do not establish causal direction or statistically different levels of node importance.

The results identify frustration-related dysregulation and negative responses to failure as candidate processes for further longitudinal, temporal, and experimental research. Exploratory differences by sex and school type require replication, and the convenience sample, common self-report method, and use of a six-node composite network constrain generalization and interpretation. Future studies using repeated measurements, multi-informant assessment, and more granular item-level networks may clarify the developmental and preventive relevance of these associations.

## Figures and Tables

**Table 1:** Sociodemographic characteristics of the participants.

	*n* (%)
Age (M ± SD)	10.27 ± 1.22
Sex	
Boys	359 (50.8%)
Girls	348 (49.2%)
School type	
Private	332 (46.9%)
Public	375 (53.1%)

**Table 2:** Descriptive measures and local network properties.

Nodes	M	SD	EI
Low frustration tolerance	16.78	5.85	1.02
Perfectionistic self-demands	15.05	4.27	0.34
Reaction to failure	15.63	4.07	0.99
Internalizing problems	4.80	2.20	0.75
Attention problems	4.25	2.27	0.59
Externalizing problems	2.61	2.16	0.58

Note. M = mean; SD = standard deviation; EI = expected influence.

## Data Availability

The individual-level dataset is not publicly available because the study involved minors and is subject to privacy and ethical restrictions. De-identified data, the analysis script, and the Spearman correlation matrix are available from the corresponding author upon reasonable request.

## Supplementary Materials

The following supporting information can be downloaded at: https://www.mdpi.com/article/10.3390/children13101411/s1, Table S1, Measurement invariance of the study instruments across sex and school type; Table S2, Simulation-based sensitivity analysis for the network comparisons.

## Abbreviations

The following abbreviations are used in this manuscript: CS, correlation stability coefficient; EBIC, Extended Bayesian Information Criterion; EI, expected influence; NCT, Network Comparison Test; PSC-17-Y, Pediatric Symptom Checklist-17, youth self-report version.
