# Supplementary material for: Low Frustration Tolerance, Childhood Perfectionism, and Mental Health Problems in Peruvian Children: A Network Analysis
Source: Children (Basel). 2026 Oct 15;13(10):1411. doi: 10.3390/children13101411 (PMC13703191; doi:10.3390/children13101411)
Supplement: Supplementary file 1 [file children-13-01411-s001.zip › children-4555133-supplementary.pdf]

Supplementary Materials

Low Frustration Tolerance, Childhood Perfectionism, and Mental Health Problems in Peruvian Children: A Network Analysis

Measurement invariance analyses. Multigroup confirmatory factor analyses were conducted across sex and school type using the weighted least squares mean and variance adjusted (WLSMV) estimator. Configural, threshold, metric, and scalar invariance models were evaluated sequentially. Changes in fit indices are reported relative to the immediately preceding model.

Table S1. *Measurement invariance of the study instruments across sex and school type.*

Grouping variable	Instrument	Model	CFI	TLI	RMSEA	SRMR	ΔCFI	ΔRMSEA	ΔSRMR
Sex	Frustration Tolerance Scale	Configural	.976	.967	.070	.052	—	—	—
Sex	Frustration Tolerance Scale	Threshold	.973	.973	.063	.052	-.003	-.007	.000
Sex	Frustration Tolerance Scale	Metric	.977	.979	.056	.055	.004	-.007	.003
Sex	Frustration Tolerance Scale	Scalar	.972	.978	.057	.059	-.005	.001	.004
Sex	Childhood Perfectionism Scale	Configural	.923	.911	.080	.083	—	—	—
Sex	Childhood Perfectionism Scale	Threshold	.923	.911	.080	.083	.000	.000	.000
Sex	Childhood Perfectionism Scale	Metric	.923	.916	.078	.083	.000	-.002	.000
Sex	Childhood Perfectionism Scale	Scalar	.921	.919	.076	.083	-.002	-.002	.000
Sex	PSC-17-Y	Configural	.938	.927	.043	.073	—	—	—
Sex	PSC-17-Y	Threshold	.938	.927	.043	.073	.000	.000	.000
Sex	PSC-17-Y	Metric	.942	.935	.041	.074	.004	-.002	.001
Sex	PSC-17-Y	Scalar	.932	.929	.043	.075	-.010	.002	.001
School type	Frustration Tolerance Scale	Configural	.980	.972	.064	.046	—	—	—
School type	Frustration Tolerance Scale	Threshold	.979	.979	.055	.046	-.001	-.009	.000
School type	Frustration Tolerance Scale	Metric	.987	.989	.041	.047	.008	-.014	.001
School type	Frustration Tolerance Scale	Scalar	.988	.990	.037	.047	.001	-.004	.000
School type	Childhood Perfectionism Scale	Configural	.921	.908	.082	.085	—	—	—
School type	Childhood Perfectionism Scale	Threshold	.921	.908	.082	.085	.000	.000	.000
School type	Childhood Perfectionism Scale	Metric	.921	.913	.080	.085	.000	-.002	.000
School type	Childhood Perfectionism Scale	Scalar	.921	.919	.077	.085	.000	-.003	.000
School type	PSC-17-Y	Configural	.929	.917	.047	.075	—	—	—
School type	PSC-17-Y	Threshold	.929	.917	.047	.075	.000	.000	.000
School type	PSC-17-Y	Metric	.933	.926	.044	.076	.004	-.003	.001
School type	PSC-17-Y	Scalar	.935	.932	.042	.076	.002	-.002	.000

Note. CFI = comparative fit index; TLI = Tucker–Lewis index; RMSEA = root mean square error of approximation; SRMR = standardized root mean square residual; PSC-17-Y = Pediatric Symptom Checklist-17, youth self-report version. Δ values represent change from the immediately preceding invariance model. All models were estimated with WLSMV for ordinal indicators.

Summary. Across all three instruments, progressively constrained models showed only small changes in CFI, RMSEA, and SRMR across sex and school type, supporting the comparability of the measurement models for the subsequent network comparisons.

Simulation-based sensitivity analysis. A simulation-based sensitivity analysis was conducted for the network comparisons by sex and school type. Using the observed subgroup-specific means and covariance structures and the actual subgroup sample sizes, 250 datasets were simulated. For each simulated dataset, the Network Comparison Test was repeated using 1,000 permutations. Empirical detection rates were calculated as the proportion of simulations yielding $p < 0.05$. Holm-adjusted detection rates account for the two grouping comparisons for the global network structure and global strength tests.

Table S2. *Simulation-based sensitivity analysis for network comparisons.*

Comparison	Detection rate	Holm-adjusted detection rate
Sex - Network structure	0.580	0.520
Sex - Global strength	0.120	0.088
Sex - RTF-IP edge	0.844	—
School type - Network structure	0.628	0.572
School type - Global strength	0.300	0.196

Note. Detection rates were obtained from 250 simulated datasets using the observed subgroup-specific means, covariance structures, and sample sizes. Each Network Comparison Test was based on 1,000 permutations. Holm-adjusted detection rates account for the two grouping comparisons (sex and school type). RTF = reaction to failure; IP = internalizing problems.
